# From denaturant to ribosome: rethinking chaperone requirements in cells

**DOI:** 10.1038/s44320-025-00167-5

**Published:** 2025-10-28

**Authors:** Yevheniia Bushman, Andrew W Truman

**Affiliations:** Department of Biological Sciences, The University of North Carolina at Charlotte, Charlotte, NC 28223 USA

**Keywords:** Proteomics, Structural Biology

## Abstract

Y. Bushman and A. Truman discuss new results by S. Fried and colleagues revealing that proteins incapable of refolding after denaturation in vitro do not reflect the requirement for chaperones for proper folding during biogenesis in vivo, in this issue of *Molecular Systems Biology*.

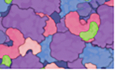

## Teaser

Traditionally, protein refolding experiments use purified proteins that are globally unfolded with chaotropic agents or heat, whereupon chaperone-mediated refolding is scored by regained structure or activity. Yadav et al profile *E. coli* proteomes lacking DnaK/DnaJ or Trigger factor and find that chaperone dependence during primary biogenesis in vivo does not track with refoldability measured in vitro. Many proteins that refuse to refold after global denaturation achieve native-like structure during co-translational synthesis, while dependence on DnaK/DnaJ varies with temperature and cofactor installation. These results reposition co-translational pathways as dominant routes to native structure and argue that client lists derived from refolding assays cannot be used as simple predictors of chaperone requirements in cells.

**Figure 1:**
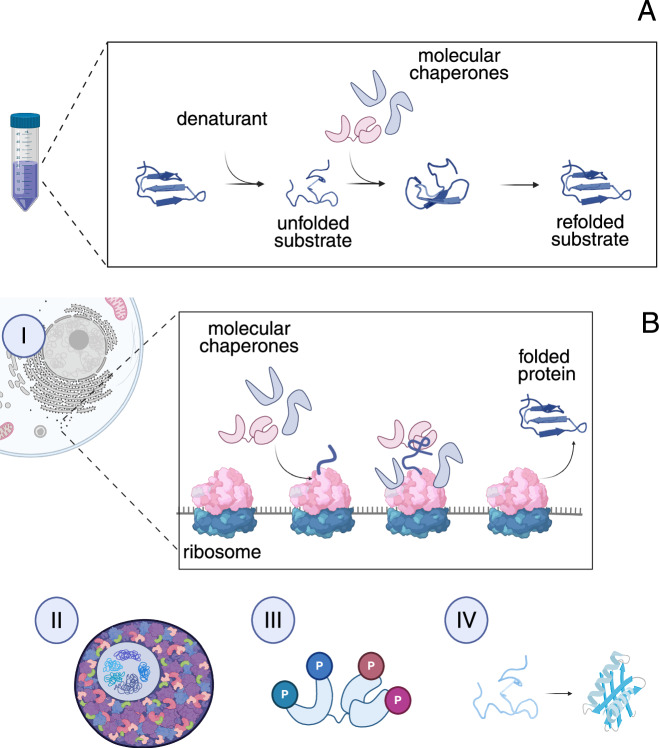
Cellular versus in vitro protein folding environments. (**A**) In vitro studies of protein folding are usually performed under denaturing conditions, where recombinant proteins are chemically or thermally unfolded and then allowed to refold, often in the presence of molecular chaperones. These experiments provide valuable mechanistic insights but lack the complexity of the cellular environment. (**B**) In vivo, protein folding occurs in a crowded and dynamic environment influenced by multiple factors, including: (i) co-translational folding as nascent chains emerge from the ribosome, (ii) macromolecular crowding that affects folding kinetics and stability, (iii) post-translational modifications that modulate chaperone activity and client interactions, and (iv) spontaneous folding of intrinsically robust proteins that require minimal chaperone assistance.

Proteins rarely fold in isolation. The crowded macromolecular cellular environment, synthesis on the ribosome, and the activity of molecular chaperones all shape a protein’s folding pathway. Chaperones support primary biogenesis at the ribosome and act post-translationally during stress by stabilizing misfolded clients and limiting irreversible aggregation. Yet much of what we think we know about chaperone-client relationships comes from canonical in vitro refolding assays, in which proteins are globally unfolded with urea, guanidinium chloride, DTT, or heat (Fig. 1A) and then mixed with chaperones while the recovery of structure or activity is measured (Haslbeck and Buchner, 2018). These experiments provided fundamental insights and defined essential roles of molecular chaperones. They also fostered a prevailing assumption: a protein’s “chaperone dependence” is an intrinsic property that should translate from the test tube to the cell. The study by Yadav and colleagues 2025 directly challenges that assumption.

The bacterial proteome is relatively simple but consists of several key chaperones, including DnaK and its co-chaperone DnaJ (Hsp70 and Hsp40 homologs) that stabilize folding intermediates, GroEL and GroES that provide a protective folding cage, as well as ATP-independent Trigger factor (Tig) that protects emerging from the ribosome chains and assists in de novo folding (Santra et al, 2017). Using limited proteolysis coupled to mass spectrometry (LiP-MS), a proteome-wide method that reports on local conformational changes by probing protease accessibility (Schopper et al, 2017), the authors investigated folding states across the *E. coli* proteome after deletion of two major chaperone systems: Tig and the DnaK/DnaJ.

Strikingly, proteins previously defined as “chaperone dependent” in refolding assays (To et al, 2021, 2022) were not consistently more destabilized in the native deletion backgrounds than those considered “independent.” In other words, in vitro refolding behavior does not reliably predict how proteins fold during primary biogenesis in the cell. Together, Tig and DnaK/J systems were thought to cover proteins that cannot reach their native states unaided. Yet Yadav et al show that many clients appear robust to the absence of individual chaperones, and that failure to refold in vitro does not necessarily equate to a requirement for chaperones in vivo. Based on their previous in vitro work (To et al, 2021, 2022), the authors focused on PGK (Phosphoglycerate kinase) enzyme. While this enzyme is considered as non-refoldable in vitro even in the presence of chaperones like DnaK/J or GroES/EL, it was structurally intact in all background in vivo in strains lacking DnaK/J or Tig. It hints that translational folding itself may be sufficient to ensure correct protein conformation (Fig. 1B). Moreover, when “chaperone dependency” was compared between in vitro and in vivo DnaK/J lacking datasets, only around 12% of proteins were considered as chaperone dependent across both datasets, highlighting a fundamental disconnect between the two contexts. Indeed, DnaK/J functions with a completely unfolded substrate, and the one emerging from the ribosome may be different.

Additional patterns appear once temperature and protein architecture are considered. Although many chaperones are induced by heat, Yadav et al show that DnaK/DnaJ also stabilizes native substrates at permissive temperature in vivo, which points to a broader housekeeping role. Trigger factor contributes more strongly at elevated temperature, yet its loss is readily buffered by other elements of the proteostasis network. Protein complexity also matters. As the domain number increases, reliance on DnaK/DnaJ increases. This dependence is most evident at 30 °C, while at 37 °C, many multidomain clients reach native structure with reduced need for DnaK/DnaJ.

Alongside well-established proteomic techniques that include cross-linking mass spectrometry and thermal proteome profiling, LiP-MS contributes to an expanding toolkit for monitoring conformational changes and chaperone effects on a proteome-wide scale (Kaur et al, 2018). These approaches bridge the long-standing gap between single-protein biochemistry and systems-level proteostasis, allowing us to uncover how proteins fold in vivo.

These advances in proteomics have also revealed a staggering number of post-translational modifications (PTMs) on Hsp70 proteins, referred to as the Chaperone Code (Nitika et al, 2020). These PTMs fine-tune chaperone function by subtly altering ATP hydrolysis, co-chaperone interaction, and client specificity. The divergence between in vitro and in vivo chaperone dependency may not only reflect the complexity of co-translational folding but also regulatory layers that modulate chaperone function depending on cellular state. Mapping these modifications in parallel with structural proteomics approaches such as LiP-MS could reveal how the chaperone code coordinates folding outcomes at the proteome scale, and how its dysregulation might contribute to proteome disturbances (Yadav et al, 2025).
